# An Exploratory Study of Transgender New Yorkers' Use of Sexual Health Services and Interest in Receiving Services at Planned Parenthood of New York City

**DOI:** 10.1089/trgh.2016.0032

**Published:** 2016-11-01

**Authors:** Lauren M. Porsch, Ila Dayananda, Gillian Dean

**Affiliations:** ^1^ Planned Parenthood of New York City, New York, New York.; ^2^ Department of Obstetrics, Gynecology and Reproductive Science, Icahn School of Medicine at Mount Sinai, New York, New York.

**Keywords:** gender nonconforming, reproductive health, sexual health, transgender

## Abstract

**Purpose:** Transgender individuals experience barriers to healthcare, including discrimination in care provision and lack of knowledge about transgender health. We assessed New York City (NYC) transgender and gender nonconforming individuals' sexual and reproductive health (SRH) needs, access to services, and interest in receiving services from Planned Parenthood of NYC (PPNYC).

**Methods:** We conducted an anonymous Internet-based survey of transgender individuals residing in NYC from September to December 2014 by using snowball sampling.

**Results:** Data were analyzed from 113 surveys. Although 74% (71/96) of respondents avoided or delayed healthcare in the past year, most respondents adhered to medically indicated SRH screenings. In the past year, 64% (45/70) and 67% (46/69) of respondents were tested for HIV and other sexually transmitted infections, respectively. In the past 3 years, 80% (39/49) of respondents received clinical breast/chest examinations and 83% (35/42) of eligible individuals received Pap tests. Respondents most often received care at LGBT specialty clinics (35% [37/105]) or at private doctors' offices (31% [32/105]). Eighteen percent (19/107) had ever been to a Planned Parenthood health center. On a four-point scale, respondents rated the following factors as most influential on whether they would seek care at PPNYC: assurance that staff received transsensitivity training (mean 3.8), the existence of gender identity nondiscrimination policies (mean 3.7), and the availability of transgender-specific services, such as hormone therapy (mean 3.7).

**Conclusions:** Although the majority of transgender individuals in our sample received recommended SRH screenings, respondents reported barriers to accessing needed medical care. Healthcare organizations interested in better serving the transgender community should ensure a high level of training around transsensitivity and explore the provision of transgender-specific services.

## Introduction

A growing body of research has demonstrated that transgender individuals in the United States face a wide range of barriers to accessing healthcare services. One pervasive barrier to healthcare for this population is discrimination based on gender identity. In community-based surveys, the prevalence of having been refused healthcare due to one's transgender status ranged from 26% of respondents in a Philadelphia area needs assessment to 39% in a San Francisco community health assessment.^1,2^ In the largest national survey of transgender individuals at the time of our online survey, the National Transgender Discrimination Survey (NTDS), 19% of respondents reported ever being denied care because of their transgender or gender nonconforming status.^3^

Additional barriers to healthcare for transgender individuals include lack of provider knowledge about transgender health issues and lack of sensitivity to transgender and gender nonconforming (TGNC) patients among providers and their staff. In a 2007 survey of transgender women in New York City (NYC), 32% of participants reported difficulty accessing a provider who was knowledgeable about transgender health issues and 30% reported difficulty accessing a provider who was transgender friendly.^4^ Correspondingly, 20% of participants in the Virginia Transgender Health Initiative Study reported that they needed to teach their primary care providers about their healthcare needs.^5^ These survey data are substantiated by qualitative research findings. A key theme that emerged from a qualitative needs assessment of transgender individuals in Minneapolis was that participants needed to regularly educate their own healthcare providers about transgender issues.^6^ Furthermore, a mixed methods study of transgender men who experienced pregnancy highlighted experiences of rude and insensitive treatment from medical staff toward transgender patients.^7^

The cost of healthcare services has also been cited as a major barrier to healthcare for transgender individuals.^4,8^ Respondents to the NTDS were less likely to have insurance coverage than the general population, and were more likely to be covered by a public insurance plan. Fifty percent of respondents to the same survey stated that they had delayed receiving necessary healthcare because of inability to afford it.^3^ Financial barriers to medical care for transgender individuals are compounded by the fact that transgender-specific healthcare services, such as hormone therapy and transition-related surgical procedures, are not covered by the majority of public and private health insurance plans.^9^

Specific areas of healthcare that transgender individuals have reported difficulty accessing include hormone therapy, transgender-related surgical procedures, preventive services, and gynecological care.^5,10^ Members of the transgender community have unique sexual and reproductive health (SRH) needs that are just beginning to be addressed in the research literature. Most of the research to date on the sexual health needs of transgender individuals has focused on HIV and sexually transmitted infections (STIs). There is a paucity of research on breast and chest health, cervical cancer screening and prevention, contraceptive needs, and providing sensitive pregnancy and abortion care for this population. Recent studies on cervical cancer screening uptake and outcomes among transgender men have demonstrated that these men are less likely to be up-to-date on Pap testing and more likely to have had an unsatisfactory Pap sample than cisgender women.^11–13^ Similarly, a recent retrospective chart review at an urban community health center demonstrated that transgender patients were less likely to adhere to mammography screening guidelines than cisgender patients.^14^ In addition, a mixed methods study of transgender men who reported sexual activity with cisgender men determined that these men had experienced pregnancy scares and accessed abortion services, and desired more information about contraceptive options and the risk of pregnancy while taking testosterone.^15^ In the first study specifically examining the experiences of transgender men who had experienced pregnancy, it was reported that one-third of the pregnancies experienced by participants were unplanned.^7^

In light of these demonstrated SRH needs among members of the transgender community, we surveyed self-identified TGNC New Yorkers about their use of SRH services and factors influencing their desire to seek care at Planned Parenthood of New York City (PPNYC). Given Planned Parenthood's expertise in providing SRH services, we wanted to better understand the factors that would affect TGNC individuals' level of comfort receiving services at a Planned Parenthood health center. Our objectives were to determine the use of SRH services among TGNC individuals in NYC, the types of services TGNC individuals in NYC would be interested in receiving at a PPNYC health center, and factors that would affect the likelihood of TGNC individuals in NYC seeking services at a PPNYC health center.

## Participants and Procedures

From September through December 2014, we conducted a cross-sectional, Internet-based survey of self-identified TGNC individuals residing in NYC. Inclusion criteria included indicating a home zip code within the five boroughs of NYC, and indicating a gender identity different from sex assigned at birth. We administered the survey through SurveyMonkey™ anonymously by disabling IP address and e-mail address tracking. The survey was determined to be exempt from IRB review by the Sterling Institutional Review Board.

While developing the survey, we contacted several community-based organizations that serve NYC's transgender community to solicit feedback on the survey instrument and request assistance in publicizing the project. After incorporating their feedback, we disseminated the survey through snowball sampling. We initiated our sampling through the social media pages and constituent listserves of PPNYC and the community-based organizations that provided feedback on the survey.

The survey consisted of 32 questions and assessed demographics, use of reproductive and sexual healthcare services, experiences of discrimination accessing healthcare, interest in receiving healthcare services from PPNYC, and factors that would enhance their comfort seeking care at PPNYC. Since some of the topics were of a sensitive nature, such as questions related to participants' sexual histories and experiences of discrimination, respondents were instructed that they could discontinue the survey at any time or skip questions. We present the number of participants who responded to each question in the Results section.

## Results

We received 149 responses, of which, 113 met inclusion criteria. Twenty-six responses were excluded because the respondents indicated a home zip code outside of NYC. Ten responses were excluded because the respondents indicated that their current gender identity was the same as the sex they were assigned at birth. Descriptive statistics were calculated for demographics and survey responses using SPSS version 22. Missing data were removed for each individual variable before analysis; thus, percentages were calculated from varying denominators depending on the number of participants who answered each question. Denominators for each variable are reported in the text and range from 113 (a question all of the 113 included respondents answered) to 12 (a question 12 respondents answered).

Seventy-three percent (82/113) of respondents were assigned female at birth (AFAB) and 27% (31/113) were assigned male at birth (AMAB). Twenty-four percent (27/113) of respondents identified as female or trans women, 35% (40/113) identified as male or trans men, and 41% (46/113) identified as genderqueer, nonbinary, or gender nonconforming. Among those AFAB, 49% (40/82) identified as male or trans men and 51% (42/82) identified as genderqueer, nonbinary, or gender nonconforming. Among those AMAB, 87% (27/31) identified as female or trans women and 13% (4/31) identified as genderqueer, nonbinary, or gender nonconforming (Table 1). Four percent of total respondents identified as black (5/113), 12% Hispanic (13/113), 76% white (86/113), 5% Asian (6/113), 1% (1/113) Native American, 8% multiracial (9/113), and 3% other (3/113). Participants could choose more than one racial or ethnic identity. Respondents' age ranged from 16 to 58 years, with a mean (±SD) age of 26 (±6.8) years. Most respondents had health insurance. Seventy-one percent of respondents (76/107) had private insurance, accessed through their employer, through a partner, through a parent, or that they had purchased for themselves. Twelve percent (13/107) had Medicaid and 2% (2/107) had Medicare. Twelve percent of respondents (13/107) were uninsured and 3% (3/107) had another type of health insurance coverage.

**Table 1.** **Gender Identity by Sex Assigned at Birth of Respondents**

	Assigned female at birth	Assigned male at birth	Total number of respondents (% of total sample)
Identified as female	0	27	27 (24)
Identified as male	40	0	40 (35)
Identified as nonbinary, genderqueer, or gender nonconforming	42	4	46 (41)
Total number of respondents (% of total sample)	82 (73)	31 (27)	113 (100)

The majority of participants reported having a regular healthcare provider, with 35% of respondents (37/105) most frequently accessing their care at an LGBT health clinic. Thirty-one percent (32/105) most frequently accessed healthcare at a private doctor or at clinician's office and 7% (7/105) received their regular healthcare at a neighborhood community health center. However, 28% of respondents (29/105) reported not having a regular healthcare provider (including those who reported using urgent care centers and hospital emergency departments as their main healthcare provider). Seventy-four percent (71/96) of respondents avoided or delayed healthcare in the past year. The reasons given for having avoided or delayed care were that the respondent could not afford to pay for care (45%, 32/71), their insurance did not cover the services they needed (44%, 31/71), they did not know where to get the services they needed (32%, 26/71), they felt that the organizations that provided the care they needed were not transgender sensitive (48%, 34/71), they were concerned that the providers would not be knowledgeable about their healthcare needs (65%, 46/71), and that they had had negative experiences receiving healthcare in the past (68%, 48/71) (Table 2).

**Table 2.** **Reasons for Avoiding/Delaying Care in the Past Year**

Reason	Percentage of those who avoided or delayed care doing so for this reason
Could not afford to pay for care	45
Insurance did not cover services needed	44
Did not know where to get services needed	32
Felt that the organizations that provided needed care were not transgender sensitive	48
Concerned providers would not be knowledgeable about transgender healthcare needs	65
Had had negative experiences receiving healthcare in the past	68

Although a large proportion of respondents had avoided or delayed accessing healthcare in the past year, at the time of the survey, most respondents had adhered to SRH-related screening recommendations (Table 3). Ninety-one percent (86/95) of respondents indicated that they had been sexually active in the past year. Sixty-four percent (45/70) had been tested for HIV and 67% (46/69) had been tested for chlamydia and gonorrhea in the prior 12 months. Seventy-three percent (70/96) of respondents indicated that they had a uterus and a cervix. Eighty-three percent (35/42) of respondents of ages 21 and older with a cervix reported that they had had a Pap test in the past 3 years. In addition, 80% (39/49) of respondents indicated that they had had a clinical breast/chest examination in the past 3 years (Table 3).

**Table 3.** **Participants' Use of Preventive Sexual and Reproductive Health Services**

Type of SRH screening^a^	Percentage of participants who received screening
Tested for HIV in the past year	64 (45/70)
Tested for other STIs in the past year	67 (46/69)
Received a clinical breast/chest examination in the past 3 years	80 (39/49)
Received a Pap test in the past 3 years (for those aged 21 years and over with a cervix)	83 (35/42)

^a^ Questions regarding the types of SRH screenings received were targeted based on the type of anatomy the respondent reported having.

SRH, sexual and reproductive health; STI, sexually transmitted infection.

Among individuals with a uterus and cervix who were sexually active in the prior 12 months, 23% (16/70) indicated that their sexual partner(s) had the ability to get them pregnant, whereas 6% (4/70) were unsure whether their sexual partner(s) had the ability to get them pregnant. Of the 20 respondents who indicated that their partner had or might have the ability to get them pregnant, only 15 responded to the question of whether they used a form of birth control to protect against pregnancy. Twelve respondents reported that they did use at least one method of contraception. The most commonly used forms of contraception were condoms (12/12), followed by oral contraceptive pills (3/12), withdrawal (3/12), or “other” (3/12) (participants were permitted to select more than one method). The methods written in by respondents under “other” included “being on testosterone” (2/12) and “avoiding penis-in-vagina intercourse” (1/12). No respondents reported using intrauterine devices (IUDs), implants, depot medroxy-progesterone acetate, the vaginal ring, the patch, or a diaphragm.

Eighteen percent (19/107) of respondents had ever been to a Planned Parenthood health center. Forty-five percent (47/105) of participants responded “yes” to the question of whether they would consider visiting PPNYC for care in future, whereas 41% (43/105) responded “maybe.” The types of services respondents were most interested in receiving from PPNYC were hormone therapy (61%), STI testing (61%), gynecological care (54%), HIV testing (52%), and breast/chest examinations (52%). A smaller proportion of participants stated that they would be interested in receiving Pap testing (38%), contraception (23%), and abortion services (19%) from PPNYC. On a four-point scale (where 1=would not influence at all and 4=would influence very much), respondents rated the influence of the following factors on whether they would seek care at PPNYC as follows: assurance that staff received transgender sensitivity training annually (mean±SD: 3.8±0.47), the existence of gender identity nondiscrimination policies (3.7±0.66), the availability of transgender-specific services, such as hormone therapy (3.7±0.70), the presence of TGNC staff (3.6±0.79), the availability of patient advocates for TGNC clients (3.5±0.79), and the availability of financial assistance and/or assistance applying for insurance programs (3.2±1.02) (Table 4).

**Table 4.** **Relative Influence of Various Factors on Participants' Willingness to Seek Care at Planned Parenthood of New York City (Where 1=Would Not Influence at All and 4=Would Influence Very Much)**

Factor	Mean (SD)
Assurance that staff receive transgender sensitivity training annually, at a minimum	3.8 (0.47)
Availability of transgender-specific services such as hormone therapy	3.7 (0.70)
Existence of gender identity nondiscrimination policies	3.7 (0.66)
Presence of TGNC staff	3.6 (0.79)
Availability of patient advocates for TGNC clients	3.5 (0.79)
Availability of financial assistance and/or assistance applying for insurance programs	3.2 (1.02)

SD, standard deviation; TGNC, transgender and gender nonconforming.

## Discussion

The majority of TGNC individuals in our sample received recommended SRH screenings. Sixty-four percent of our respondents had been tested for HIV in the past year, which is twice the proportion of NYC adults who had had an HIV test in the past 12 months according to the New York City Department of Health and Mental Hygiene's (NYC DOHMH) 2014 Community Health Survey.^16^ Among those eligible for a Pap test in our sample, 83% had received one in the past 3 years. This finding is similar to the prevalence of being up-to-date on Pap screening in the general New York State population, which the Kaiser Family Foundation reported as 88% in 2009.^17^ Although rates of preventive SRH screenings were comparable to or better than those of general populations, where comparison data existed, a very high proportion of our sample reported facing barriers to healthcare that resulted in delaying or avoiding the receipt of services. Seventy-four percent of our respondents reported delaying or avoiding healthcare in the past year, which is substantially higher than the 9.6% of adult NYC residents who reported not getting needed medical care in 2014 on the NYC DOHMH Community Health Survey.^18^ Our participants' most commonly cited reasons for delaying or avoiding care included having prior negative experiences in a healthcare encounter, concerns about providers' insensitivity and/or lack of knowledge about transgender health issues, and the costs associated with care. These findings are consistent with prior research demonstrating the multiple difficulties TGNC people experience when trying to obtain healthcare.^1–8^ By contrast, prior research examining reasons for delaying care in a general adult population has found that the most commonly cited reasons for delaying care to be not deeming one's medical problem serious, lack of time, difficulty getting an appointment, and the cost of care.^19^

Planned Parenthood provides sexual and reproductive healthcare to 2.5 million individuals in the United States annually at more than 650 health centers.^20^ Eighteen percent of our survey respondents had ever been to a Planned Parenthood health center, which is a similar proportion to the one in five American women who have ever received care from a Planned Parenthood health center.^20^ PPNYC undertook this survey to further our work to more sensitively and competently serve TGNC individuals in our community.

Our survey results demonstrate that TGNC individuals in our sample indicated interest in accessing a full range of SRH services at PPNYC, including STI and cancer screenings, contraception, and abortion. Among respondents who were at risk of pregnancy during the 12 months before taking the survey, the vast majority were using condoms as the sole method of birth control. Condoms are a lower efficacy contraceptive method, with an 18% failure rate over 1 year of typical use.^21^ In addition, a few respondents wrote in the “other method” field that they were using testosterone as a contraceptive method, which has not been shown to reliably suppress ovulation. This finding is consistent with emerging research demonstrating that TGNC individuals who were AFAB lack awareness about their risk for pregnancy while using testosterone.^22^ TGNC individuals who were AFAB may find a wide range of hormonal or nonhormonal contraceptive methods acceptable as long as they are provided with appropriate counseling as to possible desired or undesired side effects. For example, contraceptive methods that do not require daily attention and that cause a lightening or cessation of menses, such as progestin IUDs or implants, may be especially desirable methods for TGNC individuals who were AFAB. Furthermore, given that individuals in this community are at risk of unintended pregnancy, our findings highlight the need for providers of abortion services to be prepared to sensitively serve TGNC individuals in their practices.

In addition to reproductive and sexual healthcare, our respondents were interested in accessing transgender-specific services, such as hormone therapy, at PPNYC. In fact, the availability of transgender-specific services was one of the most highly rated factors that would increase the likelihood of our respondents seeking care at PPNYC. The TGNC individuals we surveyed wanted assurance that healthcare organizations have done the preparatory work required to competently and sensitively meet their needs. The existence of gender identity nondiscrimination policies, regular staff training in transgender sensitivity, the presence of TGNC staff, the availability of patient advocates for TGNC clients, and assistance paying for care were all rated highly as factors that would increase participants' likelihood of seeking care at our organization. These findings may be applicable to any healthcare provider seeking to serve TGNC clients.

A key part of PPNYC's mission is to provide healthcare services regardless of ability to pay, thus removing financial barriers to care for many of its clients. The organization provides assistance applying for public and private insurance, as well as sliding scale services for those who do not qualify for insurance. This type of assistance may be especially beneficial for members of the TGNC community, given that the NTDS found that transgender Americans were less likely to have health insurance coverage than the general population. The NTDS was conducted before the implementation of the Affordable Care Act (ACA); however, it is unclear to what extent the ACA has patched gaps in coverage for care for TGNC Americans. In May of 2016, the Department of Health and Human Services issued a final rule implementing Section 1557 of the ACA, which prohibits discrimination on the basis of sex in federally funded health programs, including health insurance plans that receive federal funds.^23^ Section 1557 includes gender identity-related discrimination under the umbrella of sex discrimination. Although the rule does not mandate the coverage of specific transgender-related medical services, it does prohibit the denial of healthcare services based solely on a person's gender identity or transgender status. Advocates are interpreting this to mean that an insurance company could not deny a medication or procedure to a transgender woman that is covered for a cisgender woman, for example.^24^

The rule was scheduled to go into effect in July of 2016. At this time, it is uncertain how much of an impact this nondiscrimination rule will have on access to care for the TGNC community. More research is needed to better understand this outcome, as well as continued advocacy and education to ensure that insurance providers abide by the new rule.

The limitations of this study include the fact that it was a cross-sectional survey that did not employ a probability sample. However, the exact size of the TGNC community in NYC is unknown, and thus it is not possible to definitively describe the sampling frame for this population. Snowball sampling, regarded as an ideal method to reach “hidden” populations, was used to recruit participants.^25^ In addition, there was a lack of racial and ethnic diversity of the sample, given that 76% of our respondents identified as white. Furthermore, in light of prior research findings that TGNC communities have lower rates of health insurance coverage, and the high rates of acquisition of preventive SRH screenings in our sample, our survey underrepresented those without health insurance. Thus, our results are likely not generalizable to the NYC TGNC community from the point of view of demographics. Possible barriers to having drawn a more diverse group of respondents are the fact that the survey was only available online and only available in English. Another drawback of the web-based survey design is that we were unable to seek clarification on seemingly discrepant responses to questions, or reasons for skipping questions. There was a lower response rate to questions relating to birth control use and use of healthcare services, and we gathered less data on these topics. We postulate that these questions may have been of a sensitive nature for some respondents; however, it would be interesting to further understand why these questions were skipped. Future qualitative research could be undertaken to gain a more indepth understanding of TGNC individuals' feelings about accessing SRH services, their experiences with the healthcare system, and their knowledge and attitudes about the services that are available to them and what they are looking for in a provider. This information could greatly inform improvements and advancements in healthcare provision for the TGNC population.

Among the strengths of our survey are that gender identity was assessed using a two-step question, consisting of current gender identity and sex assigned at birth. This method of soliciting sex and gender information is considered a best practice for identifying TGNC individuals in research and in clinical care.^26^ A high proportion of our participants identified as nonbinary or gender nonconforming, rather than with a binary gender identity. The specific healthcare needs of this population are poorly understood and warrant additional research. Furthermore, our survey is one of the few studies to date to ask about contraceptive use in TGNC individuals at risk of pregnancy. Our results demonstrate that many TGNC individuals are at risk of unintended pregnancy and may not have accurate information about, or access to, the most effective contraceptive methods. Future research should investigate how to best provide contraceptive services for this population.

Despite recent strides in health insurance coverage for TGNC individuals and increasing awareness of their healthcare needs, barriers to healthcare persist for this community. As healthcare coverage care expands, ongoing research will be needed to determine whether more providers are offering services to the TGNC community and whether they are providing the necessary staff and organizational training to provide them effectively. The fact that more than one-quarter of our respondents did not have a regular healthcare provider is concerning, as LGBT specialty clinics and private providers who are sensitive and knowledgeable about transgender health are not universally accessible. Therefore, more healthcare providers and organizations need to be prepared to serve this community.

## Abbreviations Used

ACA: Affordable Care Act
AFAB: assigned female at birth
AMAB: assigned male at birth
DOHMH: Department of Health and Mental Hygiene
IUDs: intrauterine devices
NTDS: National Transgender Discrimination Survey
PPNYC: Planned Parenthood of New York City
SD: standard deviation
SRH: sexual and reproductive health
STI: sexually transmitted infection
TGNC: transgender and gender nonconforming
